# Comparative Analyses of Chloroplast Genome Provide Effective Molecular Markers for Species and Cultivar Identification in *Bougainvillea*

**DOI:** 10.3390/ijms242015138

**Published:** 2023-10-13

**Authors:** Xinggu Lin, Shiou Yih Lee, Jianzhong Ni, Xiaomin Zhang, Xing Hu, Peishan Zou, Wei Wang, Guofeng Liu

**Affiliations:** 1 Guangzhou Landscape Plant Germplasm Resource Nursery, Guangzhou Institute of Forestry and Landscape Architecture, Guangzhou 510540, China; linxinggu@hotmail.com (X.L.); njz889@163.com (J.N.); zhangxmzbh@163.com (X.Z.); xingerjiayou@gmail.com (X.H.); zoupsh@mail2.sysu.edu.cn (P.Z.); waynelove@126.com (W.W.); 2 Guangzhou Collaborative Innovation Center on Science-Tech of Ecology and Landscape, Guangzhou Institute of Forestry and Landscape Architecture, Guangzhou 510540, China; 3 Faculty of Health and Life Sciences, INTI International University, Nilai 71800, Malaysia; shiouyih.lee@newinti.edu.my; 4 College of Forestry, Hainan University, Haikou 570228, China

**Keywords:** *Bougainvillea*, chloroplast genome, phylogenomics, molecular markers, genetic resources

## Abstract

*Bougainvillea* is popular in ornamental horticulture for its colorful bracts and excellent adaptability, but the complex genetic relationship among this genus is fuzzy due to limited genomic data. To reveal more genomic resources of *Bougainvillea*, we sequenced and assembled the complete chloroplast (cp) genome sequences of *Bougainvillea spectabilis* ‘Splendens’. The cp genome size was 154,869 bp in length, containing 86 protein-coding genes, 38 tRNAs, and eight rRNAs. Cp genome comparison across 12 *Bougainvillea* species (*B. spectabilis*, *B. glabra*, *B. peruviana*, *B. arborea*, *B. praecox*, *B. stipitata*, *B. campanulata*, *B. berberidifolia*, *B. infesta*, *B. modesta*, *B. spinosa*, and *B. pachyphylla*) revealed five mutational hotspots. Phylogenetic analysis suggested that *B. spectabilis* published previously and *B. glabra* clustered into one subclade as two distinct groups, sister to the subclade of *B. spectabilis* ‘Splendens’. We considered the phylogeny relationships between *B. spectabilis* and *B. glabra* to be controversial. Based on two hypervariable regions and three common plastid regions, we developed five molecular markers for species identification in *Bougainvillea* and applied them to classify 53 ornamental *Bougainvillea* cultivars. This study provides a valuable genetic resource for *Bougainvillea* breeding and offers effective molecular markers to distinguish the representative ornamental species of *Bougainvillea*.

## 1. Introduction

The *Bougainvillea* genus belongs to the family Nyctaginaceae, consisting of at least 18 species as recorded at present [1]. They are evergreen subtropical shrubs, lianas, or small trees. *Bougainvillea* is native to South America but was introduced to European and Asian countries in the mid-18th century as a potential ornamental plant. Currently, three *Bougainvillea* species, namely *B. glabra*, *B. peruviana*, and *B. spectabilis*, are ideal ornamental resources due to their colorful bracts and attractive foliage [2]. Their strong adaptability to various soil and climatic conditions makes them widely cultivated all over the world. Moreover, *Bougainvillea* spp. are prompt to natural hybridization, resulting in new hybrids with unique morphological characteristics, while artificial cross breeding, bud sports and induced mutations also produce thousands of cultivars [3,4].

During the past decades, *Bougainvillea* has been commercially well exploited and the development of its breeding programs and landscape application has overshadowed study on its genetic resources. At present, the names of most *Bougainvillea* cultivars are confused because of commercial purpose and conducts, and their genetic relationships are quite uncertain as a result of diverse breeding approaches and intricate historical parentage records. For instance, ‘Double Red’, ‘Klong Fire’, ‘Mahara’, ‘Mahara Crimson’, ‘Mahara Double Red’, ‘Manila Magic Red’, ‘Manila Red’, ‘Million Dollar’, and ‘Princess Mahara’ may refer to the same cultivar based on morphological characteristics [5]. In various literatures, ‘Pink Pixie’ (also known as ‘Pixie’, ‘Hawaiian Torch’, ‘Mini Thai’, ‘Smartipants’, and ‘Torch Glow’) has been considered to be a *B. glabra* or *B. spectabilis* cultivar or a *B. glabra* × *B. spectabilis* hybrid [4,5,6]. To a certain extent, the molecular phylogeny of *Bougainvillea*, based on isozymes [7], random amplified polymorphism DNA (RAPD) [7,8,9], and simple sequence repeat (SSR) [10], has developed an insight into species identification. However, RAPD markers were less reliable and reproducible. Previous research applied too few SSR markers and revealed limited genetic information. For these reasons, it is requisite to develop more effective genetic markers of *Bougainvillea* for facilitating species and cultivar identification, plant evolution, genetic engineering, and breeding.

In recent years, the focus on genome-scale information in plants has been increasing substantially. The chloroplast (cp) genome is a well-known informative resource to construct phylogenetic analysis and species delimitation [11]. With the advent of new sequencing techniques, thousands of cp genomes from the plant kingdom have been revealed, giving light to the Tree-of-Life at the genome-scale level [12]. Similarly, researchers conducted a quite comprehensive phylogenetic study to reveal the relationships among 25 genera of Nyctagineae in the family Nyctaginaceae and demonstrate *Bougainvillea* as sister to *Belemia* and *Phaeoptilum*, using concatenated cp sequences of protein-coding genes *ndhF*, *rps16*, *rpl16* and nrITS [13]. However, limited sampling of these genera meant that it was not possible to evaluate the phylogenetic positions within *Bougainvillea* species. Recently, cp genomes of 12 *Bougainvillea* species have been published [14,15,16,17], which are valuable data in deciphering the evolution history across *Bougainvillea* and contribute toward the species identification and genetic improvement for *Bougainvillea*. The phylogenetic reconstructions based on plastid genomes provided further insight to distinguish the relationships within the genus level of *Bougainvillea* [17] but have not focused on effective molecular markers for the *Bougainvillea* genus. Furthermore, the relationship of *B. glabra* and *B. spectabilis* presented much taxonomic confusion. *Bougainvillea spectabilis* ‘Splendens’ is a classic and ancient representative cultivar of *B. spectabilis* [18]. Unexpectedly, we found that *B. spectabilis* ‘Splendens’ was clustered with *Bougainvillea praecox* based on fragment sequences of cp genome and was located in a different clade beyond the *B. spectabilis* group (Figure S1). In addition, *B. spectabilis* has a closer relationship with *B. glabra* than with *B. spectabilis* ‘Splendens’.

In order to understand the structure and rapid evolution of the *Bougainvillea* genome and assess the interspecific relationship between *B. glabra* and *B. spectabilis*, we sequenced the cp genome of *B. spectabilis* ‘Splendens’ using next-generation sequencing techniques. Combining the cp genomes of other *Bougainvillea* species, we performed genome comparison and phylogenetic analysis to identify hypervariable regions and genetic relationships among *Bougainvillea.* Eventually, we classified 53 ornamental *Bougainvillea* cultivars with five molecular markers at the cp genome-level. These efforts will contribute toward the construction of a cp genome database for *Bougainvillea*, which will further support the development of molecular breeding strategies for *Bougainvillea* spp.

## 2. Results

### 2.1. Morphological Characteristics and Chloroplast Genome Structure of B. spectabilis ‘Splendens’

**Figure 1:**
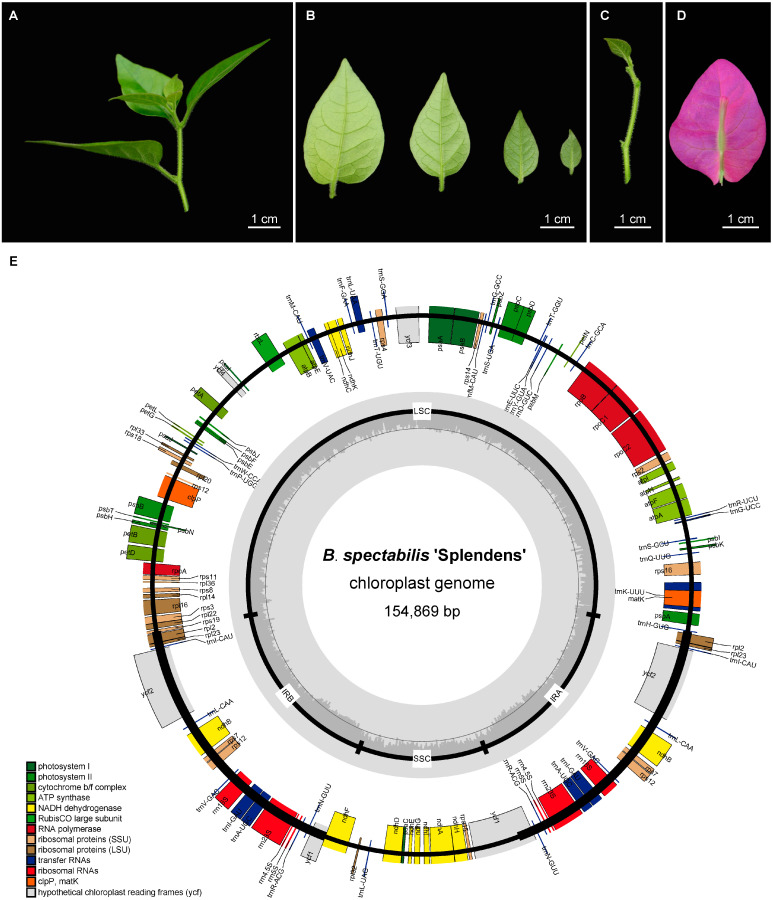
Morphological characteristics and cp genome structure of *B. spectabilis* ‘Splendens’. (**A**) Twig, (**B**) Leaves, (**C**) Stem, and (**D**) bract and perianth tube showed densely villous characteristics. (**E**) Circular gene map of the cp genome for *B. spectabilis* ‘Splendens’. Genes inside the circle were transcribed counterclockwise, and genes outside the circle were transcribed clockwise. The color-coded boxes indicate genes of different functional groups. The inner circle marks the region boundaries of LSC, IRs and SSC. The dark gray and light gray plots inside the inner circle represent GC content and AT content, respectively.

*B. spectabilis* ‘Splendens’ has densely pubescent stems, leaves and perianth tube (Figure 1A–D), the typically morphological characteristics of *B. spectabilis*. In addition, *B. spectabilis* ‘Splendens’ has a single recurved thorn in the axils of alternate leaves and a green, narrowly tubular and rounded perianth tube borne on the inner surface of elliptic-ovate fuchsia bracts, flowering during winter to spring.

**Figure 2:**
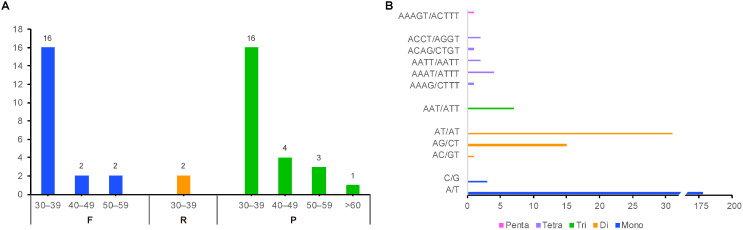
Analysis of repeat sequences and simple sequence repeats (SSRs) in *B. spectabilis* ‘Splendens’ cp genome. (**A**) Different types of repeat sequences identified in *B. spectabilis* ‘Splendens’ cp genome; F, R, and P represent forward, reverse, and palindromic repeat sequences, respectively. (**B**) Various SSRs detected in *B. spectabilis* ‘Splendens’ cp genome.

The cp genome of *B. spectabilis* ‘Splendens’ exhibits a typical quadripartite structure comprising a genome size of 154,869 bp (Figure 1E) and containing an 85,962 bp large single-copy region (LSC) and a 18,061 bp small single-copy region (SSC) separated by a pair of 25,423 bp inverted repeat regions (IRs). A total of 131 genes were predicted in the cp genome, including 86 protein-coding genes, 37 tRNAs, and eight rRNAs. The GC content for the cp genome was 36.5%. For repeat structures, we detected a total of 20 forward, two reverse, and 24 palindromic repeat sequences in the cp genome, mostly ranging from 30 to 60 bp in size (Figure 2A). Moreover, we identified 246 simple sequence repeats (SSRs), comprising 181 mononucleotides, 47 dinucleotides, 7 trinucleotides, 10 tetranucleotides, and one pentanucleotide, without hexanucleotide repeats (Figure 2B).

### 2.2. Comparative Genomic Divergence and Nucleotide Variability

**Figure 3:**
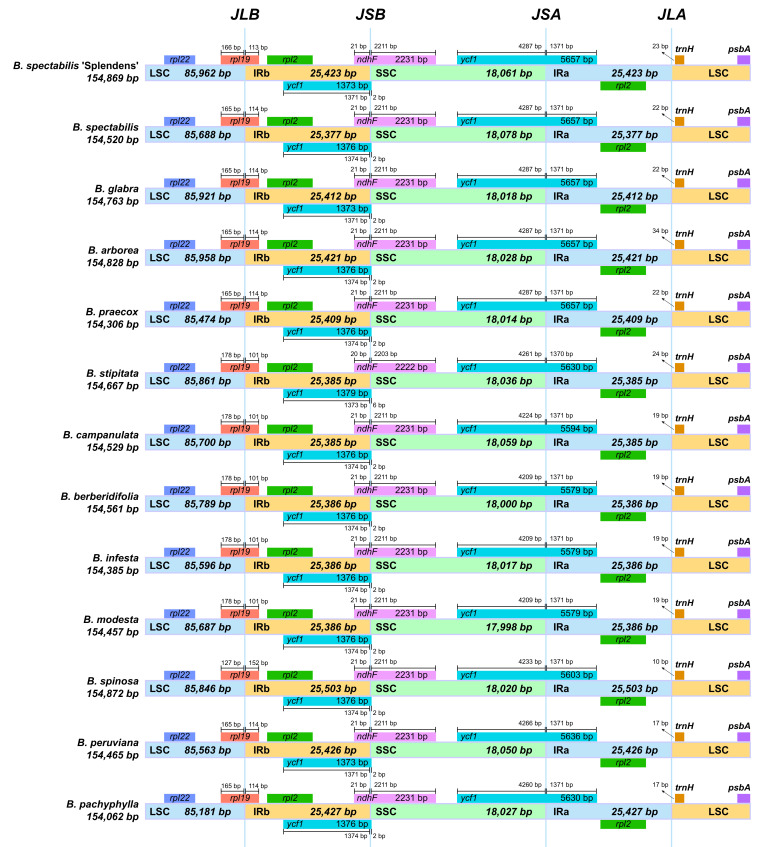
Comparisons of LSC, SSC, and IRs boundaries among 13 cp genomes within *Bougainvillea*.

The structural characteristic comparison among *B. spectabilis* ‘Splendens’ and 12 *Bougainvillea* species showed highly conserved gene contents and arrangements (Figure 3). All these *Bougainvillea* species had *rps19* and *ycf1* genes in the LSC/IRb junction region (JLB) and SSC/IRa junction region (JSA), respectively. Moreover, the *ycf1* fragment in the IRb region overlapped with the *ndhF* gene located in the junction of SSC and IRb regions (JSB). However, the sizes of the complete chloroplast genome in *Bougainvillea* were diverse, as a result of the common occurrence of contractions and expansions in the IR boundaries. Of all *Bougainvillea* cp genomes, *B. spinosa* had the longest length of IRs (25503 bp) and the largest genome size (154,872 bp), while *B. spectabilis* had the shortest length of IRs (25,377 bp).

**Figure 4:**
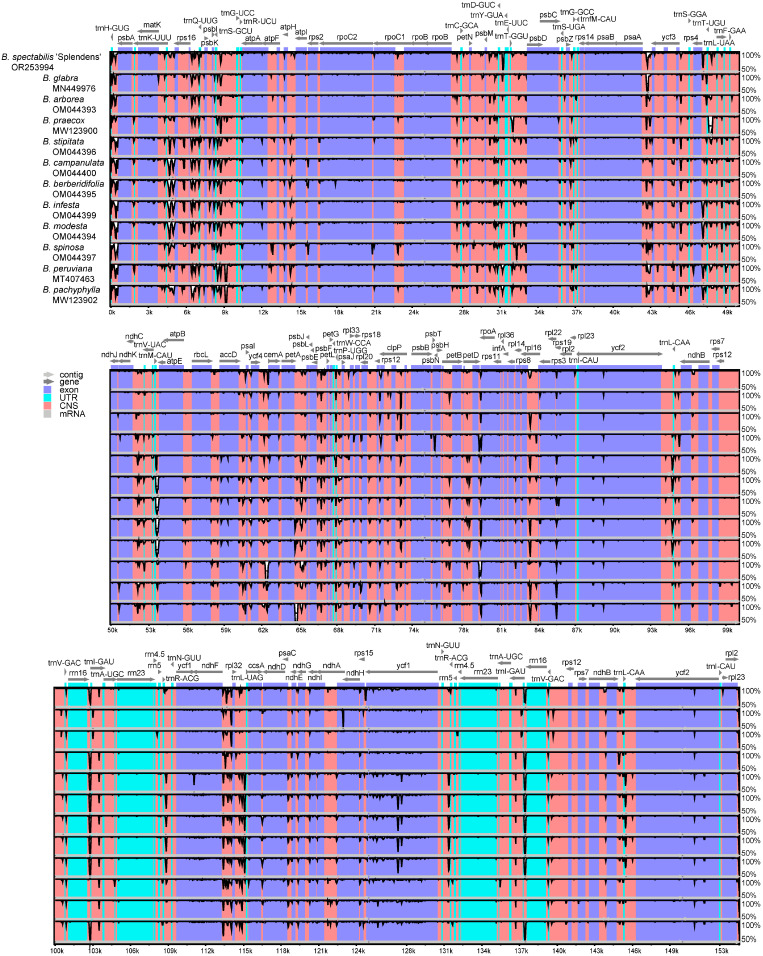
Comparisons of sequence identity for 13 cp genomes of *Bougainvillea*.

To elucidate cp genome divergence in *Bougainvillea*, we aligned and compared multiple sequences using mVISTA website (https://genome.lbl.gov/vista/mvista/submit.shtml, accessed on 9 May 2023) and setting the cp sequence of *B. spectabilis* published previously (MW167297) as the reference (Figure 4). It was evident that the non-coding regions were more variable than the coding regions. For instance, the intergenic regions of *trnH-psbA*, *trnK-rps16*, *rps16-trnQ*, *psbl-trnG*, *psbM-psbD*, *psaA-ycf3*, *trnL-trnF*, *petA-psbJ*, *ndhF-rpl32*, and *rpl32-trnL* exhibited high divergence. Furthermore, the *ycf1* protein-coding gene showed obvious variations in *B. stipitata*, *B. campanulata*, *B. berberidifolia*, *B. infesta*, and *B. modesta* when compared to the other species of *Bougainvillea*.

**Figure 5:**
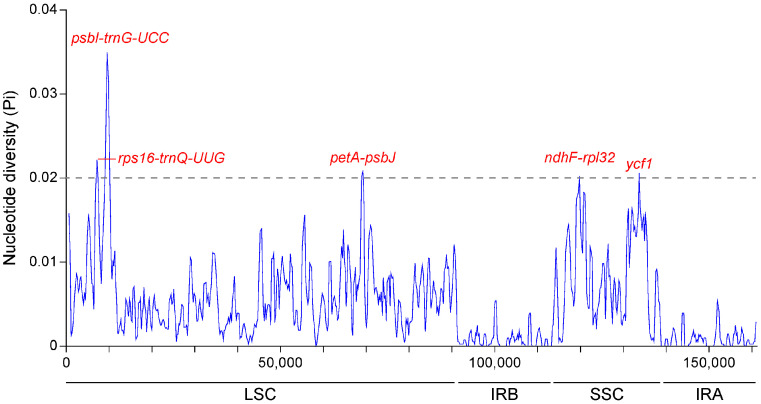
Comparison of potential mutational hotspots in the complete chloroplast genomes among *Bougainvillea*.

In order to further determine hypervariable regions of *Bougainvillea*, we compared the nucleotide diversity (Pi) values across the whole-chloroplast genomes. Among these *Bougainvillea* species, the sliding window analysis revealed five divergent hotspots with Pi values ranging from 0.020 to 0.035, including *rps16-trnQ*, *psbl-trnG*, *petA-psbJ* in the LSC region, and *ndhF-rpl32*, *ycf1* in the SSC region (Figure 5). In particular, the intergenic region of *psbl-trnG* showed the highest Pi value of 0.035, well above the average Pi value of 0.0048. Significantly, no mutational hotspots were detected in the two IR regions, further confirming the highly conservative level of IR regions in *Bougainvillea*. Moreover, we compared the numbers of SNP sites and Gaps to determine the characteristics of these five highly variable regions with *B. spectabilis* ‘Splendens’ as the reference (Table 1 and Table 2) and found the largest number of SNPs to be located in the *psbl-trnG* region. In contrast to *B. spectabilis* and *B. glabra*, *B. praecox* had the least number of SNP sites, ranging from 6 to 20, indicating that *B. spectabilis* ‘Splendens’ has a closer relationship with *B. praecox*. All these variances of chloroplast genomes provided potential molecular markers of species identification in *Bougainvillea*.

### 2.3. Phylogenetic Analysis

**Figure 6:**
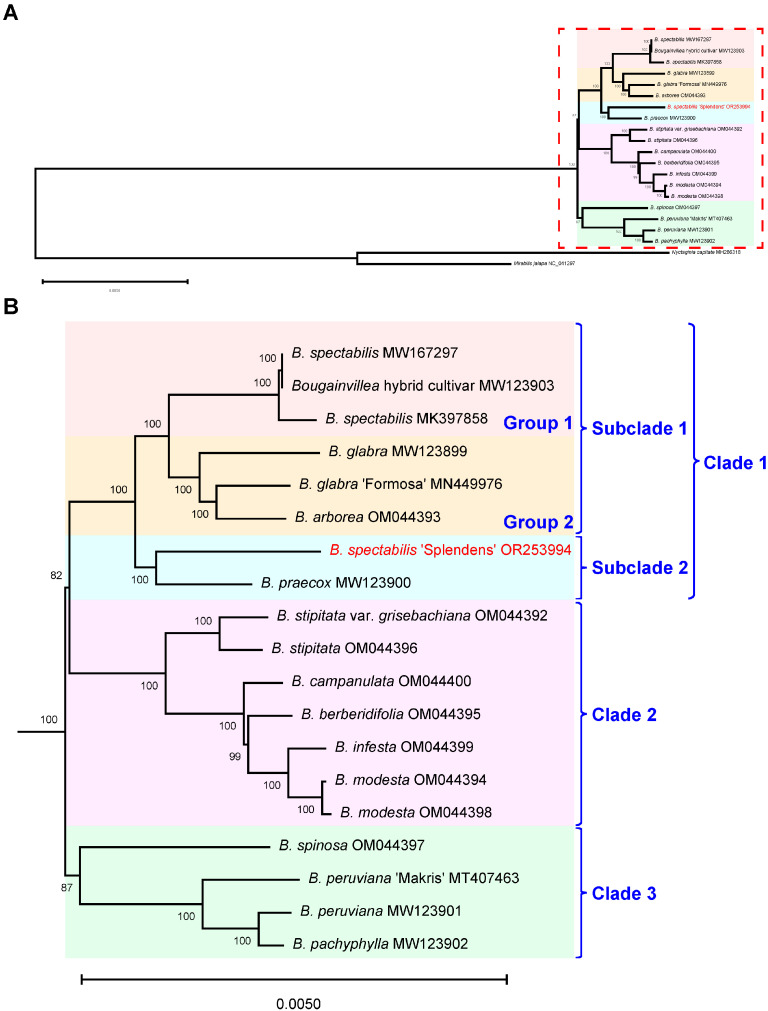
Maximum Likelihood (ML) tree inferred from the complete cp genomes of 19 *Bougainvillea* plants and 2 Nyctaginaceae species. Numbers at the nodes represent bootstrap values. (**A**) Complete graph of ML tree. (**B**) Enlarged view of ML tree in the red box of Figure 6A.

The chloroplast genomes have conserved the structure and appropriate rate of nucleotide evolution, provide unique genetic information, and lay the primary foundation for the current framework of plant phylogenetic relationships [19]. In order to clarify the phylogeny of *Bougainvillea* species, we constructed an ML tree based on a total of 19 complete chloroplast genomes, including 12 species, 4 cultivars and 1 variety (Figure 6). The phylogenetic tree revealed that *B. spectabilis* ‘Splendens’ was closely related to *B. praecox*, located in the same subclade (Subclade 2). Surprisingly, *B. spectabilis* published previously and *B. glabra* formed one subclade as two distinct branches (Group 1 and Group 2), then became sister to the subclade of *B. spectabilis* ‘Splendens’. Moreover, compared to *B. spectabilis*, *B. glabra*, and other species in *Bougainvillea*, the cluster of *B. peruviana* and *B. pachyphylla* appeared to represent the basal taxa (Clade 3), the earliest *Bougainvillea* species diverged from the Nyctagineae tribe. In general, approximately all the nodes received high support rates, ranging from 82% to 100%, indicating that the topological structure of the phylogenetic tree had high credibility.

**Figure 7:**
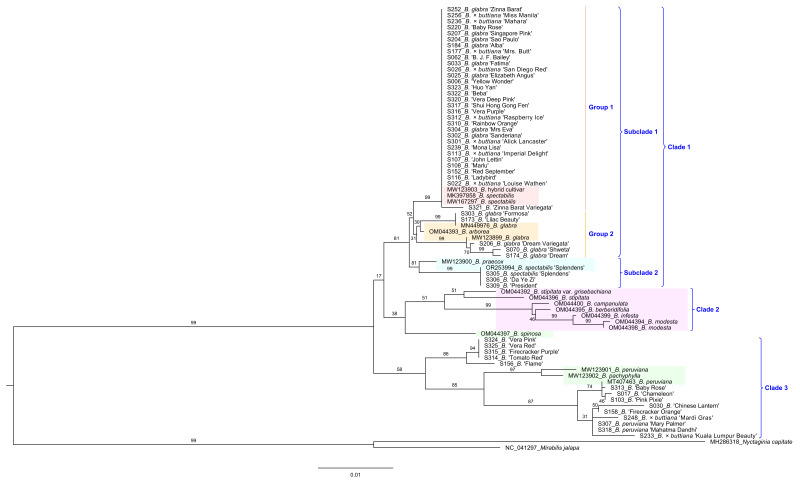
ML tree analysis of 53 *Bougainvillea* cultivars and 12 representative *Bougainvillea* species based on the combination of *trnH-psbA*, *trnL* intron, *trnL-trnF*, *psbl-trnG*, and *petA-psbJ* sequences. The five background colors indicate different clusters from the ML tree of complete cp genomes (Figure 6).

In order to identify different cultivars of *Bougainvillea*, we constructed a phylogenetic analysis of 53 cultivars based on three plastid regions, *trnH-psbA*, *trnL intron*, and *trnL-trnF*, which were commonly applied to distinguish the genetic backgrounds of diverse species [20,21,22,23]. The result showed that these three regions did not evaluate the species classification clearly (Figure S1), compared with the topological tree of *Bougainvillea* (Figure 6). Therefore, we further explored the relationships within these *Bougainvillea* cultivars based on an additional two hypervariable regions, *psbl-trnG* and *petA-psbJ.* The concatenated sequences of these five molecular markers demonstrated favorable discriminating capabilities to clarify the different taxa of *Bougainvillea* (Figure 7), which was basically consistent with the topological structure constructed by the whole-genome sequences (Figure 6), except for the position of *B. spinosa*. As expected, the majority of *Bougainvillea* cultivars were derived from the three ornamental species, *B. glabra*, *B. spectabilis*, and *B. peruviana.* In addition, we found that most cultivars were clustered with *B. spectabilis* published previously (Figure 7, Group 1). Obviously, *B. glabra* ‘Dream’ (S174), *B. glabra* ‘Dream Variegata’ (S206), *B. glabra* ‘Shweta’ (S070), *B. glabra* ‘Formosa’ (S303), and *B.* ‘Lilac Beauty’ (S173) were located at the *B. glabra* subclade (Figure 7, Group 2). Notably, *B. spectabilis* ‘Splendens’ (S305) was closely related to *B.* ‘President’ (S309) and *B.* ‘Da Ye Zi’ (S306), which formed a sister branch with *B. praecox* (Figure 7, Subclade 2). Distinct from *B. glabra* and *B. spectabilis*, ‘Baby Rose’ (S313), ‘Chameleon’ (S017), ‘Pink Pixie’ (S103), ‘Chinese Lantern’ (S030), ‘Firecracker Orange’ (S158), *B.* × *buttiana* ‘Mardi Gras’ (S248), *B. peruviana* ‘Mary Palmer’ (S307), *B. peruviana* ‘Mahatma Dandhi’ (S318), and *B.* × *buttiana* ‘Kuala Lumpur Beauty’ (S233) were clustered with *B. peruviana* (Figure 7, Clade 3). Another group, including *B.* ‘Vera Pink’ (S324), *B.* ‘Vera Red’ (S325), *B.* ‘Firecracker Purple’ (S315), *B.* ‘Tomato Red’ (S314), and *B.* ‘Flame’ (S156) also had a close relationship with *B. peruviana* and *B. pachyphylla*. These results demonstrated that the five combined markers derived from chloroplast genomes offered a potential molecular tool for the origin discrimination of *Bougainvillea* cultivars.

## 3. Discussion

In this study, we reported the assembly and annotation of reference-quality cp genome for the classic and representative *B. spectabilis* cultivar ‘Splendens’, which possesses a typical quadripartite structure with a single LSC region, a single SSC region, and two IR regions, similar in cp genome size, gene number, and structure, as well as total GC content, to those other species of *Bougainvillea* [14,15,16,17]. The comparative analysis of this cp genome and previously published data of *Bougainvillea* species provided new insight into identifying polymorphic markers for variety discrimination and evolutionary studies in *Bougainvillea.*

It is known that intergenic spacer regions are hypervariable regions that were used as potential DNA markers for phylogenetic studies and species delimitation [24]. Among the *Bougainvillea* species, we found ten divergent intergenic regions, including *trnH-psbA*, *trnK-rps16*, *rps16-trnQ*, *psbl-trnG*, *psbM-psbD*, *psaA-ycf3*, *trnL-trnF*, *petA-psbJ*, *ndhF-rpl32*, and *rpl32-trnL*, with four of these regions showing a high Pi value. Especially, *trnH-psbA* has been a popular and efficient DNA barcode for taxonomic studies [25,26]. Other than *trnH-psbA*, the intergenic *trnL-trnF* has also been commonly used for analysis of phylogenetic relationships in Ranunculaceae [27] and Rubiaceae [21]. However, the combination of *trnH-psbA*, *trnL-trnF*, and *trnL intron* could not sufficiently explain interspecific discrepancies in *Bougainvillea* (Figure S1). Consequently, we selected two more hypervariable regions, *psbl-trnG* and *petA-psbJ*, to explore the genetic affiliation of different *Bougainvillea* varieties; we confirmed the availability of these plastid genomic markers. As expected, these five cp molecular markers split *Bougainvillea* species and cultivars into three clades, containing two subclades and two groups (Figure 7), showing a similar topological structure to previous research [17]. In addition, we identified the *ycf1* gene as one of the mutational hotspots (Figure 4 and Figure 5). To date, the *ycf1* protein-coding gene is considered to be the most promising core barcode of land plants at the plastid genome level [28], benefiting from remarkable variability. Similarly, the *ycf1* gene exhibited apparent differentiation in *B. stipitata*, *B. campanulata*, *B. berberidifolia*, *B. infesta*, and *B. modesta* (Figure 6, Clade -2); however, these were not the major ornamental *Bougainvillea* species. Therefore, the *ycf1* gene is not an optimum marker for distinguishing *Bougainvillea* varieties. At present, we have developed the chloroplast molecular markers of *Bougainvillea* based on the comparative analysis of whole cp genomes and confirmed that they could effectively distinguish the origin from different maternal species of *Bougainvillea.*

According to the ML tree, the famous variety ‘Sanderiana’, derived from *B. glabra* [29], seemed to have a closer relationship with *B. spectabilis* (MK397858 and MW167297) than with *B. glabra* (MN449976 and MW123899), and this group comprised many *B. glabra* and *B.* × *buttiana* cultivars (Figure 7, Group 1), which is unexpected. Based on the breeding history of *Bougainvillea*, *B.* × *buttiana* was developed from the interspecific hybridization of *B. glabra* and *B. peruviana* [4,29]. Thus, it is not surprising that the *B.* × *buttiana* cultivars ‘Miss Manila’, ‘Mahara’, ‘Mrs. Butt’, ‘San Diego Red’, ‘Raspberry Ice’, ‘Alick Lancaster’, ‘Imperial Delight’, and ‘Louise Wathen’ clustered together with the majority of *B. glabra* cultivars (Figure 7, Group 1). Moreover, it is clear that the cultivars ‘Shweta’, ‘Dream’, and ‘Formosa’ came from the maternal origin of *B. glabra* (Figure 7, Group 2), which is consistent with previous literatures [4,6,29,30]. However, it is hard to believe that *B. spectabilis* ‘Splendens’, a classic and ancient representative cultivar of *B. spectabilis* [18], was far from the group of *B. spectabilis*. On the contrary, the *B. spectabilis* group was sister to the *B. glabra* group. We assume that there are two possible reasons for this result. On the one hand, the cp genome sequences of *B. spectabilis* and *B. glabra* may be too similar to separate from each other, although *B. spectabilis* was more closely related to *B. glabra* than to *B. spectabilis* ‘Splendens’, contradicting the fact that interspecific differences should be greater than intraspecific differences. On the other hand, the previously published cp genome of *B. spectabilis* may have contained some mistakes in sampling. In morphologically taxonomic features, *B. spectabilis* has densely villous leaves and a short villous perianth tube, while *B. glabra* has merely puberulent leaves and a sparsely to densely puberulent perianth tube [17]. As a result, it may be easy to confuse these two species with only morphological distinction. Based on the origin of cultivars and phylogenetic analysis, it is reasonable to assume that the previously published cp genome of *B. spectabilis* may contain some mistakes, and they probably belonged to the *B. glabra* polyphyletic group or a hybrid population of *B. glabra* × *B. spectabilis*.

*B. peruviana* cultivars ‘Mary Palmer’ and ‘Mahatma Dandhi’, as well as *B.* × *buttiana* cultivars ‘Mardi Gras’ and ‘Kuala Lumpur Beauty’, locate at the clade of *B. peruviana*, which corresponds to their parental origin [4,5,6,29]. Nevertheless, there are also some controversial results. For instance, *B.* ‘Pink Pixie’ was closer to *B. peruviana*, according to the phylogenetic tree based on cp molecular markers, whereas it was considered to be a *B. glabra* cultivar, a *B. spectabilis* cultivar, or a *B. glabra* × *B. spectabilis* hybrid in previous literatures [4,5,6]. Besides, we found that *B.* ‘Flame’ and ‘Tomato Red’ cultivars located at a sister cluster to *B. peruviana* but were thought to have originated from *B. spectabilis* [5]. In addition, *B.* ‘Mona Lisa’ had a closer relationship with the majority of *B. glabra* and *B.* × *buttiana* cultivars, although it was previously classified as a cultivar of *B. peruviana* [6]. Furthermore, *B.* ‘President’ formed a cluster with *B. spectabilis* ‘Splendens’ based on a high supporting value, while it was considered to be a *B. glabra* cultivar in the checklist of *Bougainvillea* cultivars [5]. These results suggest that there may be quite a few errors in the records and descriptions of the origins of *Bougainvillea* cultivars. Also, although these cp genome markers can distinguish *Bougainvillea* varieties to a greater extent, identification for some varieties probably needs further exploration in the future.

## 4. Materials and Methods

### 4.1. Plant Materials and DNA Extraction

Fresh leaves of *B. spectabilis* and 53 cultivars were collected from the Germplasm Resource Nursery of Ornamental Plants (Table S1), Guangzhou Institute of Forestry and Landscape Architecture, Guangzhou, China. The leaf tissues from an individual plant were sampled for all 53 cultivars. The leaves were kept in aluminum ziplock bags and transported back to the laboratory to be kept at −80 °C prior to DNA extraction. Total DNA extraction was carried out using DN15 Plant DNA Mini Kits (Aidlab Biotechnologies, Beijing, China) according to the manufacturer’s protocol. DNA quantification and quality were estimated through Nanodrop 2000 C spectrophotometry (Thermo Fisher Scientific Inc., Waltham, MA, USA) and 1% (*w*/*v*) agarose gel.

### 4.2. Chloroplast Genome Sequencing, Assembly and Annotation

A 300 bp insert size genomic library was constructed using the TruSeq DNA Sample Prep Kit (Illumina, San Diego, CA, USA) and was sequenced on an Illumina Novaseq platform (Illumina, San Diego, CA, USA). Approximately 6 Gb of raw data of 150 bp paired-end reads were generated and were further removed for their adapter sequences using the NGS QC Toolkit [31]. Raw reads were subjected to de novo assembly using NOVOPlasty v3.8.1 [32], using the complete chloroplast genome of *B. spectabilis* (Genbank accession MW167297) as a reference and the *rbcL* gene from *B. spectabilis* as the initial seed sequence. The assembled genome was annotated using the online tools GeSeq (https://chlorobox.mpimp-golm.mpg.de/geseq.html, accessed on 12 November 2022) [33] and CpGAVAS2 (http://47.96.249.172:16019/analyzer/home, accessed on 12 November 2022) [34], then manually checked for annotation errors. The circular plastid genome map for *B. spectabilis* was visualized using OGDRAW v1.3.1 [35]. Then, the cp genome was deposited at the GenBank database with accession number OR253994. Repeat sequences containing the three types of forward, reverse, and palindromic were identified using REPuter (https://bibiserv.cebitec.uni-bielefeld.de/reputer, accessed on 20 November 2022) [36], whereby the Hamming distance was fixed at 3 and the minimum repeat size was set at 30 bp. Simple sequence repeats (SSRs) were defined using MISA-web (https://webblast.ipk-gatersleben.de/misa/, accessed on 21 November 2022) [37] with the parameters set as follows: the minimum number of repeats for mono-, di-, tri-, tetra-, penta- and hexa-nucleotides were eight, four, four, three, three, and three, respectively.

### 4.3. Genome Comparison and Variation Analysis

The newly sequenced cp genome of *B. spectabilis* was compared to 12 available cpDNA sequences of *Bougainvillea* species (Table S2), which were downloaded from the NCBI GenBank database. The expansions and contractions in the IR boundary locations of these *Bougainvillea* sequences were compared using the IRscope (https://irscope.shinyapps.io/irapp/, accessed on 23 November 2022) [38]. To detect variations within the cp genomes of *Bougainvillea*, sequence alignment was performed using the Shuffle-LAGAN mode of mVISTA (https://genome.lbl.gov/vista/index.shtml, accessed on 27 November 2022) [39], and the whole-chloroplast sequence of *B. spectabilis* published previously (MW167297) was applied as the reference. To identify the hypervariable regions among the 12 representative species of *Bougainvillea*, the cp genomes were aligned using MAFFT v7.487 [40], then nucleotide diversity (Pi) values were calculated using DnaSP v6.12.03 [41], utilizing sliding window analysis with a window length of 600 bp and a step size of 200 bp.

### 4.4. Phylogenetic Analysis

For phylogenetic tree construction, 19 cp genome sequences from 12 species of *Bougainvillea*, downloaded from NCBI, were included in the maximum likelihood (ML) analysis. Two closely related Nyctaginaceae species, *Nyctaginia capitate* (MH286318) and *Mirabilis jalapa* (NC_041297), were included as an outgroup. Genome sequence alignment was carried out using MAFFT v7.487. The ML tree was constructed using MEGA 7 (v11.0.10) based on the Kimura 2-parameter (K2P) model with 1000 bootstrap replications. The resultant Newick tree document was visualized under Figtree v1.4.4 (http://tree.bio.ed.ac.uk/software/figtree/, accessed on 29 November 2022).

### 4.5. Primer Design and PCR Amplification

Based on conserved nucleotide sequences at both ends of mutation hotspots, 5 pairs of specific primers were designed to identify 53 cultivars of *Bougainvillea* using Primer Premier 5 (Table S3). The PCR reaction mixture consisted of 1.25 μL of genomic DNA, 12 μL of 10× LA PCR Buffer, 1 μL of dNTP Mix, 2 μL of forward and reverse primers (10 μmol/L), 0.5 μL of LA Taq (Takara, Osaka, Japan) and ddH_2_O supplemented to 25 μL. PCR amplification was performed under the following conditions: a single initial denaturing stage at 94 °C for 5 min; subsequently 40 cycles for denaturation, annealing, and extension reactions set at 94 °C for 30 s, 56–60 °C for 30 s, and 72 °C for 30 s, respectively; then a final extension at 72 °C for 20 min. Fragment lengths of the PCR products were examined by electrophoresis on 1% agarose gel and visualized with 4S GelRed Nucleic Acid Stain (Sangon Biotech, Shanghai, China). Finally, the sequences of PCR products were analyzed on an ABI PRISM 3730XL Genetic Analyzer (Applied Biosystems).

### 4.6. Multiple Sequence Alignment and Effectiveness of Marker Discriminatory

The forward and reverse sequencing data was assembled by the SeqMan module of DNAStar v7.1.0 (http://www.dnastar.com/, accessed on 11 December 2022). The assembled fragment sequences were aligned using MAFFT v7.487 according to similarity of base composition. For detecting the resolution and effectiveness of chosen markers, single and combination sequences of five selected variation regions were applied to construct the ML and Neighbor-joining (NJ) trees based on 1000 bootstrap replications using the K2P model of MEGA 7 (v11.0.10) [42].

## 5. Conclusions

Our results clearly describe the cp genome characteristics of *B. spectabilis* ‘Splendens’ and provide insights into the cp genome divergence and phylogenetic evolution of *Bougainvillea* species. The high resolution of the ML tree suggests that the cp genome is a powerful tool to resolve the phylogeny relationships at the *Bougainvillea* genus level. In order to accurately illustrate the genetic affiliation of representative cultivars from *Bougainvillea*, we developed five molecular markers to effectively distinguish the main ornamental species of *Bougainvillea.* These results serve as an important reference for *Bougainvillea* breeding, especially overcoming the cross-incompatibility among extensive varieties of *Bougainvillea*.

## Figures and Tables

**Table 1:** Multiple analysis of the mutational hotspots in LSC region within 13 *Bougainvillea* plants.

Species	Mutational Hotspots											
	*rps16-trnQ-UUG* Position: 6744–7587				*psbI-trnG-UCC* Position: 8957–9978				*petA-psbJ* Position: 68,799–69,549			
	Length	GC Content	Number of SNPs	Number of Gaps	Length	GC Content	Number of SNPs	Number of Gaps	Length	GC Content	Number of SNPs	Number of Gaps
*B. spectabilis* ‘Splendens’ OR253994	723	23.7%	/	/	748	22.3%	/	/	628	28.3%	/	/
*B. spectabilis* MW167297	712	23.2%	23	11	738	22.1%	20	10	636	27.4%	11	−8
*B. glabra* MN449976	701	24.4%	17	22	776	21.4%	12	−28	635	28.0%	6	−7
*B. arborea* OM044393	688	24.6%	16	35	794	21.5%	12	−46	635	28.2%	9	−7
*B. praecox* MW123900	724	23.6%	16	−1	692	23.7%	7	56	633	28.3%	6	−5
*B. stipitata* OM044396	713	24.4%	26	10	798	21.2%	45	−50	684	26.0%	20	−56
*B. campanulata* OM044400	704	24.6%	29	19	745	22.3%	35	3	702	26.4%	21	−74
*B. berberidifolia* OM044395	710	24.4%	28	13	745	22.1%	34	3	672	27.1%	21	−44
*B. infesta* OM044399	692	24.9%	24	31	743	22.3%	37	5	684	26.8%	21	−56
*B. modesta* OM044394	705	24.4%	28	18	751	22.0%	36	−3	684	26.8%	21	−56
*B. spinosa* OM044397	703	23.8%	20	20	752	21.8%	44	−4	684	28.4%	19	−56
*B. peruviana* MT407463	713	23.6%	30	10	716	22.5%	60	32	628	27.7%	18	0
*B. pachyphylla* MW123902	710	23.7%	30	13	779	21.4%	62	−31	628	27.9%	17	0
Total	/	/	287	/	/	/	404	/	/	/	190	/

**Table 2:** Multiple analysis of the mutational hotspots in SSC region within 13 *Bougainvillea* plants.

Species	Mutational Hotspots							
	*ndhF-rpl32* Position: 119,304–120,150				*ycf1* Position: 133,364–134,041			
	Length	GC Content	Number of SNPs	Number of Gaps	Length	GC Content	Number of SNPs	Number of Gaps
*B. spectabilis* ‘Splendens’ OR253994	701	20.4%	/	/	636	22.3%	/	/
*B. spectabilis* MW167297	734	20.3%	23	−33	636	22.6%	10	0
*B. glabra* MN449976	686	21.1%	22	15	636	22.6%	14	0
*B. arborea* OM044393	686	21.0%	12	15	636	23.0%	14	0
*B. praecox* MW123900	672	20.4%	20	29	636	22.2%	7	0
*B. stipitata* OM044396	748	19.7%	23	−47	657	21.8%	16	−21
*B. campanulata* OM044400	764	19.6%	26	−63	621	22.2%	19	15
*B. berberidifolia* OM044395	746	19.7%	25	−45	621	22.5%	14	15
*B. infesta* OM044399	765	19.6%	23	−64	621	22.5%	15	15
*B. modesta* OM044394	746	19.7%	23	−45	621	22.5%	15	15
*B. spinosa* OM044397	737	20.2%	36	−36	636	22.5%	12	0
*B. peruviana* MT407463	739	20.3%	30	−38	642	22.4%	16	−6
*B. pachyphylla* MW123902	740	20.3%	34	−39	642	22.7%	14	−6
Total	/	/	297	/	/	/	166	/

## Data Availability

The assembled chloroplast genome sequence of *Bougainvillea spectabilis* ‘Splendens’ has been deposited in GenBank under accession number OR253994.

## Supplementary Materials

The supporting information can be downloaded at: https://www.mdpi.com/article/10.3390/ijms242015138/s1, references [43,44,45,46] are cited in Supplementary Materials.
