# Supplementary figures and images for: Comparative Analyses of Chloroplast Genome Provide Effective Molecular Markers for Species and Cultivar Identification in Bougainvillea
Source: Int J Mol Sci. 2023 Oct 13;24(20):15138. doi: 10.3390/ijms242015138 (PMC10607086; doi:10.3390/ijms242015138)

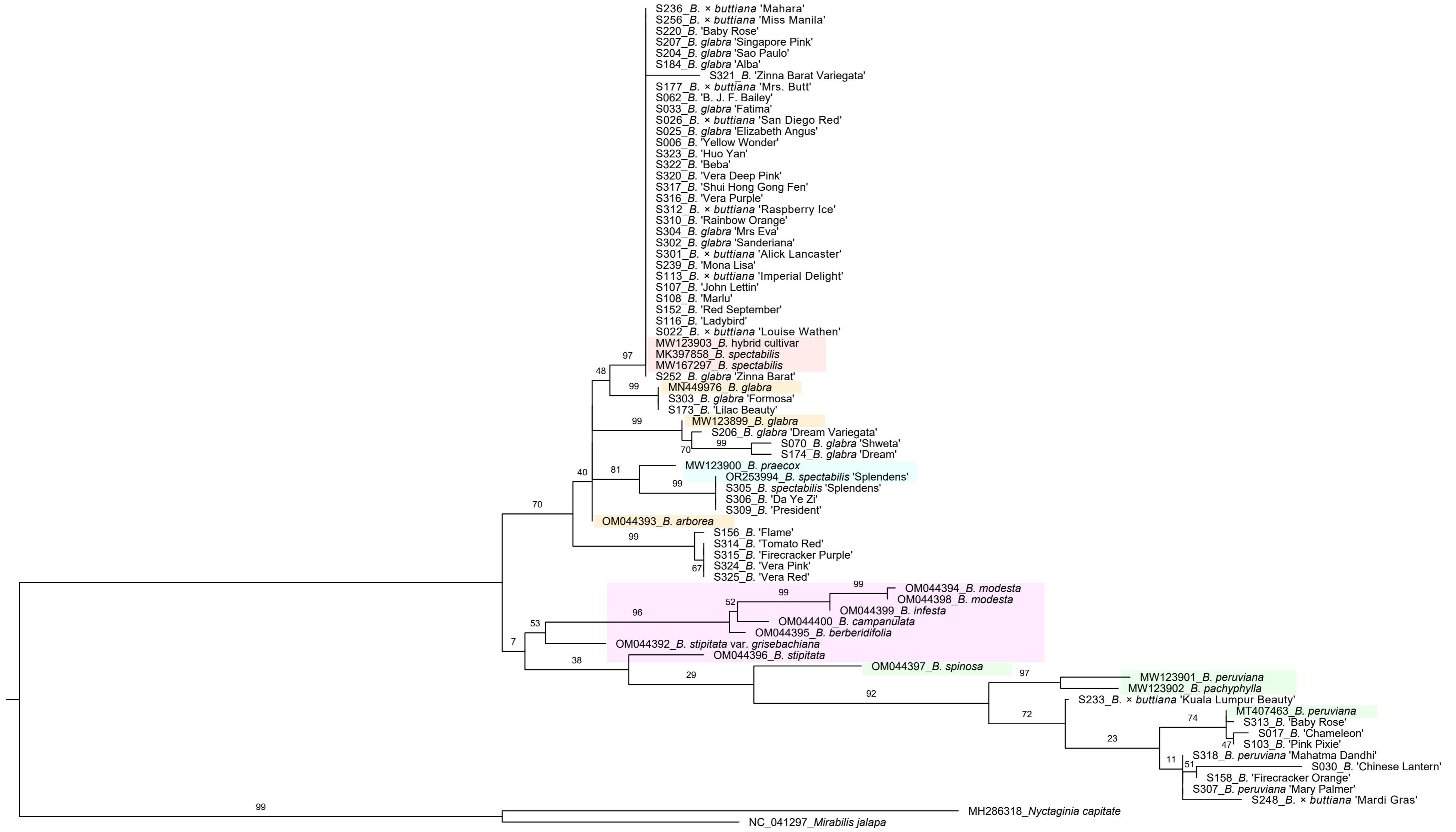

0.02

Supplement: Supplementary file 1 [file ijms-24-15138-s001.zip › supplementary files_resubmit/Figure S1.pdf]
